# Efficacy of an educational intervention in primary health care in inhalation techniques: study protocol for a pragmatic cluster randomised controlled trial

**DOI:** 10.1186/s13063-016-1269-5

**Published:** 2016-03-17

**Authors:** José Leiva-Fernández, Rubén L. Vázquez-Alarcón, Virginia Aguiar-Leiva, Mireya Lobnig-Becerra, Francisca Leiva-Fernández, Pilar Barnestein-Fonseca

**Affiliations:** Vélez Sur Primary Care Centre / IBIMA Institute, Málaga Este-Axarquía Health Area / Málaga University, Vélez Málaga, Málaga, Spain; Multiprofessional Family and Community Attention Teaching Unit, Málaga-Guadalhorce Trust, Málaga University, Málaga, Spain; Multiprofesional Family and Community Medicine Attention Teaching Unit, Málaga-Guadalhorce Trust, Málaga University, Málaga, Spain; Multiprofessional Family and Community Attention Teaching Unit / IBIMA Institute, Málaga-Guadalhorce Trust / Málaga University, Málaga, Spain

**Keywords:** COPD, Inhalation techniques, Educational intervention, Primary care professionals

## Abstract

**Background:**

Chronic obstructive pulmonary disease (COPD) accounts for 10–12 % of primary care consultations, 7 % of hospital admissions and 35 % of chronic incapacity related to productivity. The misuse of inhalers is a significant problem in COPD because it is associated with reduced therapeutic drug effects leading to lack of control of both symptoms and disease. Despite all advice, health care professionals’ practice management of inhalation treatments is usually deficient. Interventions to improve inhaler technique by health care professionals are limited, especially among primary care professionals, who provide the most care to patients with COPD. The aim of this study is to evaluate the efficacy of an educational intervention to train general practitioners (GPs) in the right inhalation technique for the most commonly used inhalers.

**Methods/design:**

We are conducting a pragmatic cluster randomised controlled trial. The sample population is composed of 267 patients diagnosed with COPD using inhalation therapy selected from among those in 20 general practices, divided into two groups (control and intervention) by block randomisation at 8 primary care centres. The sample has two levels. The first level is patients with COPD who agree to participate in the trial and receive the educational intervention from their GPs. The second level is GPs who are primary health care professionals and receive the educational intervention. The intervention is one session of the educational intervention with a monitor given to GPs for training in the right inhalation technique. The primary outcome is correct inhalation technique in patients. Secondary outcomes are functional status (spirometry) and quality of life. The follow-up period will be 1 year. GPs will have two visits (baseline and at the 1-year follow-up visit. Patients will have four visits (at baseline and 3, 6 and 12 months). Analysis will be done on an intention-to-treat basis.

**Discussion:**

We carried out three previous clinical trials in patients with COPD, which showed the efficacy of an educational intervention based on monitor training to improve the inhalation technique in patients. This intervention is suitable and feasible in the context of clinical practice. Now we are seeking to know if we can improve it when the monitor is the GP (the real care provider in daily practise).

**Trial registration:**

ISRCTN Registry identifier ISRCTN93725230. Registered on 18 August 2014.

## Background

Chronic obstructive pulmonary disease (COPD) accounts for 10–12 % of primary care consultations, 35–40 % of hospital consultations, 7 % of hospital admissions and 35 % of disability related to productivity [1]. The disease cost represents 2 % of the annual budget of the Ministry of Health and Consumer Affairs in Spain [1], and in the European Union the total costs of respiratory disease are estimated to be about 6 % of the total health budget; 56 % of this cost is due to COPD [2]. Two thousand euros per year has been estimated as the mean direct cost generated by these patients. The major part (43.8 %) corresponds to hospital admissions, followed by controlled drug consumption (40.8 %) in patients with mild COPD. This percentage decreases to 37.4 % and 28.4 % in patients with Global Initiative for Chronic Obstructive Lung Disease (GOLD) stages III and IV, respectively [3].

The administration of drugs using inhalation technologies has changed the treatment of respiratory diseases. These drugs have advantages over systemic therapy, including faster delivery to the targeted organ and reduction of side effects [4]. The effectiveness of inhaled drugs can be influenced by numerous factors, which are mainly age, sex, education level, years of diagnosis, type of inhaler, use of the right inhalation technique or use of different devices [5, 6].

The misuse of inhalers is a significant problem in asthma and COPD because it is associated with reduced therapeutic drug effects leading to a lack of control both symptoms and disease [7–10]. If patients are prescribed a treatment without proper training, a less than optimal therapeutic effect results [10, 11].

Even though clinical practice guidelines advocate the implementation of health education programmes during medical follow-up, in some studies researchers have observed that more than 85 % of patients do not correctly use their inhalers [11–14]. The educational interventions proposed by these guidelines must include aspects such as positive reinforcement, smoking cessation advice, diet and exercise information, adherence to the therapeutic regimen and verification of the right inhalation technique [15, 16].

International guidelines on the management of COPD refer to the following aspects related to the inhalation technique [2, 17, 18]. (1) The effectiveness of the bronchodilator therapy should be assessed on the basis of not only the pulmonary capacity of the patient but also other aspects, such as improvement of symptoms, improvement in daily activities, capacity to perform exercise and self-control of the symptoms. (2) Most of the patients, independently of age, can learn to use the proper inhalation technique with appropriate training. (3) Once the patient has shown the ability to perform the right inhalation technique, the device should be prescribed. (4) The patient’s ability to use devices must be checked regularly by highly trained health care professionals, and, if necessary, the patient must be retrained in the proper inhalation technique.

Despite these recommendations, the use of inhaler devices by health care professionals is deficient [10, 19]. Interventions to improve inhalation technique in health care professionals are limited, especially among primary health care professionals, who provide care to most patients with COPD [19, 20].

### Study aims

Our research group has studied the management of chronic illness in primary care for more than 15 years, focusing mainly on COPD. The first step taken was to carry out trials to develop tools to assess therapeutic adherence and educational interventions to improve adherence. Our first educational intervention was designed with three components (motivational and cognitive aspects, as well as skill development in inhalation techniques). The intervention was evaluated in a randomised controlled trial which showed that treatment adherence improved by 48 % (relative risk) with a number needed to treat (NNT) of 6.37 [14]. In the last 3 years, our clinical trials have been focused on the evaluation of educational interventions to improve inhalation techniques in patients with COPD. We have designed two educational interventions to improve inhalation technique: (1) only written information (leaflet) or (2) written information (leaflet) reinforced by instructor training. These trials showed that an intervention with a demonstration by a trainer is significantly more effective than an informative leaflet alone, with an increase in the number of patients who develop proper inhalation technique by 48 % at 3 months and by 41 % at 1-year follow-up [21].

The next step is the start-up of an intervention focused on primary health care professionals so that they are well-trained. The main goal is to evaluate the efficacy of an educational intervention to train general practitioners (GPs) in the right inhalation technique for the most commonly used inhalers and, afterwards, to evaluate the improvements in inhalation techniques performed by their patients. The secondary outcomes of this intervention will be a clinical control improvement and follow-up of the disease as well as improvement in health-related quality of life. All of these outcomes have been key goals for more than 10 years.

### Study hypothesis

On the basis of current published evidence, inhalation techniques in patients with COPD are insufficient, and this overlaps with a lack of well-trained health care professionals. Our working hypothesis is that providing an educational intervention to primary care professionals (GPs) will increase by at least by 25 % the number of patients who report use of the correct inhalation techniques compared with a control group of patients receiving standard care.

## Methods/design

### Overview

**Fig. 1:**
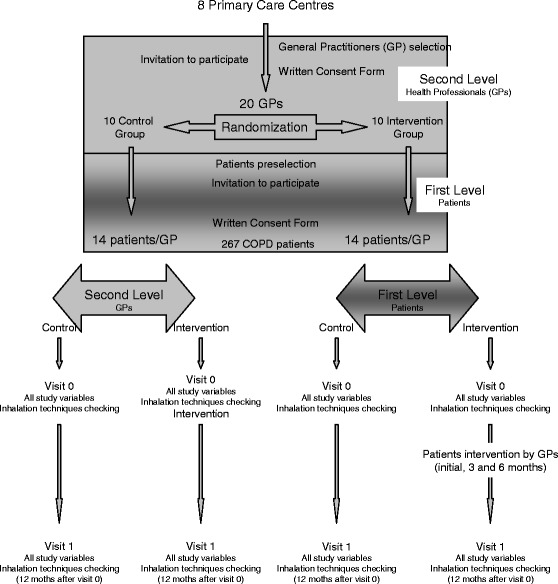
Study scheme. *COPD* chronic obstructive pulmonary disease

This study is a pragmatic cluster randomised controlled trial (Fig. 1). There are various circumstances in which the pragmatic cluster randomised controlled trial has shown to be the most appropriate design, particularly when health programmes are more focused on the organisational level. This design can also avoid “contamination” between patients when an educational intervention is applied in a primary care centre, owing to its having been individually randomised. This contamination is due to communication between participants, which can change the real effect of the intervention. Finally, when educational strategies for the right patient control are focused on health care professionals, the only adequate strategy is the randomised allocation of them [22, 23].

For our trial, the cluster design is based on two levels: the higher or second level, represented by the GP (who will receive the educational intervention), and the lower or first level, represented by the GP’s patients (who have agreed to participate and will receive the educational intervention from their GP).

### Participants

A total of 267 patients diagnosed with COPD and receiving inhalation therapy will be selected from 20 general practices in 8 primary care centres in Málaga and Almería, Spain. This sample size is enough to detect a 25 % difference between groups regarding the right performance of inhalation technique, with a power of 80 % and a confidence level of 95 %. The sample size was adjusted according to the standard criteria for cluster randomised trials, using the design effect (DE) of 2.3. The DE was calculate as follows: DE = 1 + (*n*_c_ – 1) × ICC (where *n*_c_ is the mean number of individuals in the cluster and ICC is the intra-cluster correlation coefficient). The ICC in the present work was considered to be 0.1, and the mean cluster size was assumed to be 10 [24–26]. A potential loss of 40 % was estimated. Therefore, inclusion of 267 patients and 20 GPs will be required, with all of them signing consent forms to participate in the study.

#### First-level participants: patients

##### Inclusion and exclusion criteria

The inclusion criteria are a COPD diagnosis, being treated at the primary care centres included in the trial, being prescribed inhalation therapy and consenting to participate in the trial by signing the informed consent form. The exclusion criteria are having another respiratory illness not included in the COPD definition and having cognitive impairments which prevent the individual from completing the outcome questionnaires or engaging with the educational intervention.

##### Recruitment

For recruitment, potentially eligible patients will be identified through a search of practise electronic records by research assistants. Eligible patients will receive a brief explanation of the intent of the study by phone, and, if they are interested, they will be given an appointment at their primary care centre for a more detailed explanation. Once the consent form has been signed, the patient’s inclusion visit will be carried out and all the variables will be measured (including the evaluation of the inhalation technique of all inhalers the patient uses). For eligible patients who decline to participate, basic information (age, sex, reason for declining) will be collected from the practice to allow examination of response bias.

##### Follow-up

The follow-up of patients will be limited to 1 year after the inclusion visit by the research team. During the inclusion visit, all variables will be measured again, including the evaluation of inhalation techniques. All measurements will be taken by a research assistant who has no knowledge of either the randomisation of the GPs or whether the patients belong to the study or control arm.

##### Interventions in patients

The patients included in the intervention group will receive an educational intervention from their GPs, who will train them in the correct use of their devices. This intervention will consist of the following:

1. Performance of inhalation techniques to detect mistakes that will be registered on a specially designed sheet
2. Demonstration of proper inhalation techniques
3. Identification of mistakes in technique and opportunities to ask health professionals about how to perform inhalation properly

The patients will be given an appointment for reinforcement visits at 3 and 6 months after the inclusion visit. In these visits, the GPs will encourage and refresh their performance of inhalation techniques.

As the educational intervention will not be blinded, the external researcher responsible for collection of patient data will not know the patient allocation. The patients included in the control group will follow the standard clinical practice.

#### Second-level participants: GPs

##### Inclusion and exclusion criteria

The inclusion criteria for second-level participants are that they must be doctors who care for patients included in the COPD Process of Andalusian Health Service Guidelines (COPD PAI) [16], use prescribed inhalation therapy and have consented to participate. The exclusion criteria are refusal to participate in the trial or having to leave the job during the trial.

##### Recruitment and randomisation

For the selection of GPs, the research project will rely on pre-selected primary care centres. Twenty GPs will be selected by using a non-probabilistic consecutive sampling method: 10 GPs as the control group and 10 GPs as the study group. To do that, GPs will be invited to participate and will be included in either group by using a block randomisation technique. Each block is formed by four GPs among whom the two study arms will be uniformly distributed. Once the blocks have been created, they will be distributed using a sequence of random numbers and the final list of GP allocations will be created. The final list of GP allocations will be guarded by the principal investigator of the project, and he will inform the allocation of each GP included in the trial.

The GP’s inclusion visit will be carried out once the randomisation has been done. In this visit, all of the study’s variables are collected and the inhalation technique of each inhaler is evaluated through a specific step-by-step test. This test was specially designed for this study on basis of the Sociedad Española de Neumología y Cirugía Torácica (SEPAR) recommendations [4] for most of the commonly used devices (Spiriva HandiHaler, Boehringer Ingelheim, Ridgefield, CT, USA; Seretide Accuhaler, GlaxoSmithKline, Philadelphia, PA, USA; turbuhalers; pressurized metered-dose inhalers; and Breezhaler, Novartis, Basel, Switzerland).

##### Follow-up

Both groups of GPs will be followed up 1 year after the inclusion visit. In this visit (visit 1), all variables will be collected once again, and the GPs use of the inhalation technique will be evaluated.

##### Interventions in GPs

The intervention consists of a demonstration of correct inhalation technique and the rationale for it to a group of two to four GPs. After the demonstration, the participants are asked to identify their mistakes and ask any questions they may have, which will be resolved until full understanding of the technique is achieved. The intervention will take approximately 15 minutes to deliver. Reinforcement with written information is provided via the patient’s data collection form (DCF), where the specific step-by-step schedule for each device is recorded at each patient’s visit (initial visit and at 3 and 6 months).

Blinding of the intervention for GPs will not be possible for either researchers or GPs. Table 1 provides a brief summary of the interventions for the first- and second-level participants.

**Table 1:** Brief summary of interventions in first and second levels

	Second level: professionals (GPs)	First level: patients
Content	Inhalation technique workshop:	Inhalation technique workshop:
	• Step-by-step correct technique demonstration (how)	• Step-by-step correct technique demonstration (how)
	• Explanation of each step for each device (why)	• Explanation of each step for each device (why)
Who applies the intervention	Research team	GPs from the intervention group
Who receives the intervention	GPs from the intervention group	Patients with COPD from the intervention group

*COPD* chronic obstructive pulmonary disease, *GP* general practitioner

### Setting

The setting is eight primary care centres in Málaga and Almería, Spain.

### Study variables

#### Individual variables/first-level variables (patients)

The primary outcome is performance of the correct inhalation technique by patients (evaluated through a specific step-by-step test for each inhaler). This test has been specially designed for this study on the basis of SEPAR recommendations [4]. We consider the correct inhalation technique to be used when all steps have been performed correctly.

Secondary outcomes are functional status as measured by forced spirometry [27], dyspnoea index as measured using the Basal Dyspnea Index [28] and the modified Medical Research Council dyspnoea scale [29] and health-related quality of life as measured using the St George’s Respiratory Questionnaire (SGRQ) [30].

Independent variables are age, sex, education level, co-morbidities, COPD diagnosis duration, mental and/or cognitive status (Mini Mental State Examination [31]), types and number of inhalers, previous training received in use of the inhalation techniques and prescribed treatment for COPD.

#### Group variables/second-level variables (GPs)

Group and second-level variables are correct performance of the inhalation techniques by GPs (evaluated using a specific step-by-step test for each inhaler, described below), knowledge about COPD and its treatment (evaluated by using a questionnaire specially designed for this study and based on COPD PAI [16], Spanish COPD Guidelines [15] and GOLD guidelines [2]).

#### Independent variables

The independent variables are age, sex, education level and access to the COPD clinical practice guidelines.

### Statistical analysis

#### General

A descriptive statistical analysis will be performed for all study variables. We will calculate the means, medians and standard deviations for quantitative variables and the absolute and relative frequencies for qualitative variables. The 95 % confidence interval will be applied. The analysis will be done according to the intention-to-treat principle. The baseline comparison will be made between the main variables that we expect to be related to the primary outcome using the χ^2^ test or analysis of variance (ANOVA).

At the first level, the between-group comparison for the primary outcome will be explored using the χ^2^ test. Relative risk reduction, absolute risk reduction and NNT will be calculated. Inferences for secondary outcomes will be made using ANOVA or the χ^2^ test.

Finally, a logistic regression model will be used for the primary outcome (performance of the right inhalation technique [yes or no]), considering the intervention as the predictive variable and the rest of the independent measures as the possible modifying factors. We will use the usual 5 % significance level (α = 0.05) and the SPSS version 15.0 statistical software package (SPSS, Chicago, IL, USA) to run the proposed analysis.

#### Multi-level analysis

ICCs will be determined and published for all primary outcome variables to assist future research. Potential confounders will be compared between groups to confirm that the randomisation has provided the appropriate balance.

The effect of the intervention on outcomes measured on a continuous scale (such as SGRQ score) will be estimated and tested using mixed model ANOVA in which time and treatment group will be fixed effects and GP and subject will be random effects. The effect of the intervention on the dichotomous variables will be analysed using generalised estimating equations with a logistic link and a model structure that is analogous to that described above.

### Study limitations

The first limitation is about the actual study design. In pragmatic cluster randomised controlled trials, the first bias to take into account is the selection bias, as the randomisation is usually applied in the second level, before recruitment of the first-level participants, which is where the intervention impact is measured. However, there are strategies that minimise this bias, and we are using these in this study. First, the selection and follow-up of patients (patient inclusion visit and visit 1) will be carried out by an external researcher who will have no knowledge of GP randomisation. Second, the effect of the intervention over recruitment can lead to different recruitment indexes, depending on the groups, due to the possibility that GPs in the control group could be less motivated to participate. Moreover, to avoid different recruitment indexes in both groups, once the trial is finished, the GPs in the control group will be trained in the same way as the study group professionals were trained (if the intervention shows a significant improvement in patients) [23, 32].

Another study limitation is the potential for loss to follow-up. To minimise these losses, patients will be contacted at least three different times and at different times of day. However, loss to follow-up was taken into account in the sample size calculation and will be taken into account in performing data analysis (intention-to-treat analysis).

Another bias is the Hawthorne effect, which relates to how participants in a study change their behaviour under observation. However, we believe that the effect of this bias will be minimal because of patients’ being trained and followed by their GPs, with the exception of the inclusion and final visits. Besides, there is a control group in which the follow-up will permit us to minimise the real impact of this bias.

There could be measurement bias due to mistakes in recording of some variables or interviewer bias due to the administration of the questionnaires. To minimise these biases, interviewers will have been trained previously to ensure that visits will be as homogeneous as possible. A DCF and a manual that explains how each variable is measured at the follow-up visits will be given to the GPs in the intervention group.

Finally, we point out that a rescue mechanism is contemplated for the control group. This mechanism will be applied when the patients perform critical errors in the inhalation techniques that compromise the totality of the drug deposition and its effect. This rescue mechanism can create an information bias which could change the effect of the educational intervention in this study. However, we must ensure the well-being of the patient. All adverse events will be reported on the DCF and will be taken into account in the post hoc analysis.

### Ethics

This study has been approved by the local ethics committee of Malaga (CEI Provincial de Malaga; 12/12/13). Patients and GPs will sign informed consent forms.

## Discussion

In the last 20 years, multiple studies have been carried out showing that health care professionals prescribe inhalers without adequate knowledge about their use. Current guidelines on treating COPD recommend that physicians demonstrate to patients how to use the different devices at their first appointment, and then to correct improper use at follow-up visits [1, 2]. However, this approach is not widely applied in routine clinical practice, as shown by the high percentage of health care professionals who are incapable of simply demonstrating the correct use of the inhaler devices [10, 20]. This lack of knowledge has not improved recently, despite efforts by health care education institutions [20].

It is widely accepted that poor disease control among patients with COPD is due to improper use of inhalation medicines [8]. The inhalation device may affect inhaler technique because there are a wide range of inhaler devices, some of which are easier to use than others [17, 19]. So, these potentially confounding factors will be considered in our strategy of statistical analysis.

Mishandling of inhalation devices decreases patient adherence to the inhaler therapeutic regimen, compromising treatment efficacy [7]. If the proposed educational intervention is effective, it stands a good chance of being implementable, owing to its simplicity. It could be exported to any health care field for inpatients and outpatients, both nationally and internationally, with adaptation for the most commonly used inhalers in each area. Previously conducted clinical trials by our research group have shown that a simple educational intervention is altogether more effective in teaching patients with COPD about the right management of their inhalers. This proposed clinical trial will verify and confirm our previous proof-of-concept work which indicated that a simple educational intervention delivered by GPs in primary care can improve inhaler technique among patients with COPD. This would provide high-quality evidence about educational interventions with primary health care professionals to improve the management of inhaler treatment, the clinical outcomes and health-related quality of life of patients with COPD, all of which are key aims in any assistance process. These factors have high importance in primary care, where most patients with COPD are treated with this kind of technique.

In addition to patient benefit related to implementation of the inhalation technique intervention (better treatment adherence, improvement in clinical status and health outcomes), we expect the intervention to be cost-effective, too, with reduction in the direct and indirect costs associated with the management of COPD. This will have a positive impact in organisations, resource management, health care services and health politics, and overall in the health and well-being of patients with COPD.

### Trial status

We have divided the participant recruitment into two phases and are recruiting GPs and patients for the second phase of the study.

## Abbreviations

ANOVA: analysis of variance
COPD: chronic obstructive pulmonary disease
COPD PAI: COPD Process of Andalusian Health Service Guidelines
DCF: data collection form
DE: design effect
GOLD: Global Initiative for Chronic Obstructive Lung Disease
GP: general practitioner
ICC: intra-cluster correlation coefficient
NNT: number needed to treat
SEPAR: Sociedad Española de Neumología y Cirugía Torácica
SGRQ: St George’s Respiratory Questionnaire
